# Vertebrate paired fins are specified by an ancient dorso-ventral patterning system

**DOI:** 10.1371/journal.pbio.3004062

**Published:** 2026-11-10

**Authors:** Rebecca E. Dale, Silke Berger, Sara Alaei, Marcus C. Davis, Frank J. Tulenko, Peter D. Currie

**Affiliations:** 1 Australian Regenerative Medicine Institute, Monash University, Clayton, Victoria, Australia; 2 Peter MacCallum Cancer Centre, Melbourne, Victoria, Australia; 3 Department of Physical and Biological Sciences, Western New England University, Springfield, Massachusetts, United States of America; 4 Victoria Node, EMBL Australia, Monash University, Clayton, Victoria, Australia; University of California San Diego, UNITED STATES OF AMERICA

## Abstract

The origin of paired fins was a major event in early vertebrate history that fueled the adaptive radiation of the gnathostome clade. Evidence for the conservation of ancient regulatory systems between paired and median fins supports a model in which appendage-patterning mechanisms first evolved in the midline and were later redeployed to a new embryonic context, the flank. However, while paired fins and limbs are asymmetric along the dorsoventral (DV) axis, the equivalent axis does not exist in median fins, which are symmetric across the midline, raising questions of how DV patterning evolved. Here, we combine genetic tools in zebrafish with comparative expression analyses in representative ray-finned and cartilaginous fishes to show that a subset of limb DV patterning genes are expressed during median fin development. Using genetic lineage tracing of the canonical limb dorsalizing factor *lmx1bb*, we further demonstrate that, although *lmx1bb*-derived cells occupy distinct spatial domains across fin types, a subset of this lineage is fated to differentiate into fin ray osteoblasts in both paired and median fins. To test the potential conservation of regulatory modules across fin types, we generated a series of deletion mutants for two known *lmx1b* mouse limb enhancers, *LARM1* and *LARM2,* that we show are partially conserved upstream of zebrafish *lmx1bb*. These experiments revealed that *LARM* function is essential for dorsoventral patterning of the paired pectoral and pelvic fins of zebrafish but is dispensable for formation of the unpaired dorsal and anal fins. Collectively, these data support the conservation of an ancient *LARM*-Lmx1b dorsal determination system in the paired fins of osteichthyans, challenge models proposing simple co-option of a pre-existing median fin gene regulatory network and suggest that the acquisition of this regulatory module may have been a key early step in the evolution of the paired fin DV patterning axis.

## Abstract 2

Appendage-patterning mechanisms first evolved in median fins along the midline, but it remains unclear how paired fins aquired their dorso-ventral patterning. This study shows that paired fins and limbs use a conserved developmental module for DV patterning that does not operate in median fins, arguing against simple network co-option.

## Introduction

The presence of an appendicular system—whether it be fins for swimming, limbs for running, or wings for flying—is one of the most recognizable features of the jawed vertebrate body plan. Classical hypotheses propose that paired fins first evolved as either transformed gill arches [1], or as retained regions of an elongate ancestral fin that extended along the trunk [2–4]. While new fossil discoveries have invigorated the debate around these hypotheses and shed light on how the vertebrate body plan was assembled (e.g., [5,6]), the origin of paired fins remains poorly understood. The earliest known vertebrate fossils possessed median fins but lacked paired fins [7–10]. While a variety of paired, lateral fin-like structures were present in jawless vertebrates, the earliest antero-posteriorly restricted paired fins that are often considered homologous to those of jawed vertebrates appeared at the pectoral level in osteostracans, whereas pelvic fins arose later in stem gnathostomes (reviewed in [11–13]; but see also [14]).

Changes in developmental programs underpin the emergence of novel morphologies. For the origin of the vertebrate paired fins, this transition would have required lateral plate mesodermal cells of the flank to acquire a novel role in producing fin outgrowths [15–18]. Molecular developmental studies on paired fin origins have provided key insights into the evolution of competency zones for appendage formation [19,20], generative homologies between limbs and gill arches [21–24], the regional patterning of lateral plate mesoderm to form a fin-forming field [15,18,25–28], and the early evolution of paired fin musculature [16,29–34]. Given that median fins predate the evolutionary origin of paired fins, a prominent developmental model for the origin of paired fins posits that pre-existing gene regulatory networks were co-opted from the midline to the flank [35,36]. Evidence in support of this model includes the similar expression profiles of 5′ *HoxD* and *Tbx* genes in paired and median fins [36], and the conservation of Shh-mediated regulatory networks for anterior-posterior polarization and outgrowth [37–40], including the ZRS *Shh* enhancer [41]. Less, however, is known about the evolution of other patterning axes. Here we focus on the evolution of the appendage dorsoventral (DV) patterning system. While paired fins and limbs have clear top (dorsal) and bottom (ventral) halves, median fins, which emerge from a median fin-fold [42], exhibit left-right mirror symmetry and lack a patterning axis that is topologically equivalent to the DV axis of paired appendages. This raises fundamental questions around how the DV patterning axis arose during vertebrate appendage evolution.

In tetrapods, a core set of molecular interactions pattern limbs along the DV axis [reviewed in [43–45]]. En1, a transcriptional repressor protein present in the ventral ectoderm of limb buds, restricts the expression of the secreted signaling molecule Wnt7a to the dorsal ectoderm [46–49]. Wnt7a, in turn, induces the expression of Lmx1b, a transcription factor, in the dorsal limb bud mesenchyme, which regulates the formation of dorsal musculoskeletal and integumentary structures, as well as motor axon pathfinding [48–58]. In mice, the loss of *lmx1b* produces a bi-ventralized limb phenotype most apparent in the autopod [43], whereas ectopic expansion of *lmx1b* expression into the ventral limb bud is sufficient to result in double-dorsalized limb morphologies [48,49,51,59,60]. In mice, Chip-seq experiments have identified two *cis*-regulatory modules, *LARM1* (containing both enhancer and silencer elements) and *LARM2*, that regulate *lmx1b* expression in developing limbs through a positive autoregulatory feedback loop and restrict expression to the dorsal limb compartment [61,62]. Notably, deletion of *LARM1-LARM2* recapitulates the bi-ventralized limb phenotype of *lmx1b* null mutants without causing negative pleiotropic effects in other tissue types, indicating the limb specificity of the *LARM* enhancer landscape [61].

Less is known about appendage DV patterning systems in finned vertebrates. Dorsoventral asymmetries have been reported in the fin rays (rod like bones that support the distal fin) of the Australian lungfish (*Neoceratodus forsteri*), the non-teleost actinopterygians *Polypterus ornatipinnis* and *Acipenser brevirostrum,* and tetrapodomorph fossils bridging the fin-to-limb transition [63]. In zebrafish, keratinized epidermal structures, termed breeding tubercles, are unique to the dorsal side of the pectoral fins of males and function in reproduction [64,65]. Molecular studies in zebrafish show that *Wnt7a* and *En1* orthologues compartmentalize the ectoderm along the DV axis, similar to limbs [66–70], and that their expression depends on *fgf10-*mediated signaling [70]. Additionally, *lmx1b* expression has been reported in the dorsally positioned adductor muscles of pectoral fins based on in situ hybridization [71]. Because fin myofibres derive from migratory somitic myoblasts [29,72], these observations contrast with those of limbs, where *lmx1b* is expressed in the lateral plate mesoderm and functions non-cell autonomously in muscle patterning [58,73]. Among cartilaginous fishes, *En1* is expressed in the ventral ectoderm of the pectoral fins of the catshark *Scyliorhinus canicula* [74], and *lmx1b* in the dorsal mesenchyme of the pectoral and pelvic fins of the little skate *Leucoraja erinacea* [75]. Collectively, these data suggest a *lmx1b*-mediated dorsoventral patterning system was already in place in the paired fins of the last common ancestor of jawed vertebrates. Most recently, the expression of dorsoventral patterning genes has been characterized in both the paired and unpaired fins of the direct-developing cichlid *Astatotilapia burtoni*, the sturgeon *Acipenser baerii,* and the catshark *Scyliorhinus caniculi* [76]. In addition to revealing a limb-like profile of *wnt7a, en1a*, and *lmx1b* in paired fins, these analyses demonstrated broad ectodermal *wnt7a* and posteriorly restricted *lmx1b* expression in the dorsal and anal fins [76]*.* Interestingly, pharmacological perturbation experiments in *A. burtoni* provide evidence that Shh- but not Wnt-mediated signaling is required for *lmx1b* expression in the median fins, suggesting the evolution of novel regulatory inputs for dorsal patterning in paired fins [76].

Here we use zebrafish genetics and comparative expression analyses to test predictions of the median fin co-option model with respect to the evolution of the “DV” patterning axis. Using new transgenic tools in zebrafish, we demonstrate that the *lmx1b* lineage contributes to both paired and median fins—forming uniquely regionalized domains that differ between fin types—and that a subset of *lmx1bb-*positive cells differentiates into fin ray osteoblasts. To functionally test the conservation of *lmx1b* regulation between fin types in zebrafish, we use enhancer deletions in cis to a *lmx1bb* knock-in fluorescent reporter to show that *LARM* enhancers are principal drivers of *lmx1bb* expression in both the pectoral and pelvic fins but do not contribute to median fin expression. Furthermore, homozygous loss of *LARM1–LARM2* results in a bi-ventralized fin ray phenotype in the paired fins that corresponds to *lmx1bb* lineage contributions but does not affect median fin morphology.

## Results

### *lmx1bb* BAC demonstrates regionalized expression in both the paired and median fins of zebrafish

To characterize the distribution and fate of cells expressing the limb dorsalizing factor *lmx1bb* during fin development in zebrafish, we first generated a stable BAC line [TgBAC(*lmx1bb*:KalTA4_mCherry)^CH211-81G2^] that included ~57 kb of sequence 5′ to the *lmx1bb* start site, which was used to drive an mCherry-KalTA4 cassette (S1A Fig). Crossing this line with a compound transgenic line that included a KalTA4 responsive mCherry reporter (UAS-Elb:Eco nfsb-mCherry) and ubiquitously expressed GFP (*ubi*:Switch) resulted in embryos with mCherry fluorescence that recapitulated a subset of sites of endogenous *lmx1bb* expression, including the midbrain-hindbrain boundary [77,78], otic capsules [71,77], and pectoral fin buds [71] (Figs 1A, 1B, S1B and S1C). In rendered coronal sections, mCherry labeling was restricted to the dorsal compartment of the pectoral fin, demonstrating that the BAC promoter was sufficient to organize regionalized fin expression (Fig 1C). As previous reports for mice and zebrafish suggest *lmx1bb* is expressed in different mesodermal lineages [58,71,73], we crossed our TgBAC(*lmx1bb*: KalTA4_mCherry)^CH211-81G2^ (UAS-Elb:Eco nfsb-mCherry) [hereafter referred to as BAC(*lmx1bb*:mCherry)] line with either a TgBAC(*pax3a*:GFP) [79,80] or TgBAC(*tbx5a:*GFP) [81,82] line to label migratory somitic myoblasts or fin-field lateral plate mesoderm, respectively. At 48hpf, *lmx1bb*:mCherry colocalized with *tbx5a*:GFP in cells of the endoskeletal disc and cells surrounding the dorsal muscle anlagen but was absent from *pax3a*:GFP positive myoblasts (Fig 1D–1K), thus confirming their lateral plate identity. To further characterize the expression profile of core components of the limb DV patterning axis in pectoral fins, we performed in situ hybridization for zebrafish orthologues of *wnt7a* and *en1*, the upstream canonical regulators of *lmx1b* in limbs. Similar to developing limbs, *wnt7aa* and *en1a* expression was confirmed in the dorsal and ventral pectoral fin ectoderm, respectively (S2 Fig). Together, these observations support the conservation of DV patterning gene spatial domains between teleost paired fins and limbs [66–71,83].

**Fig 1:**
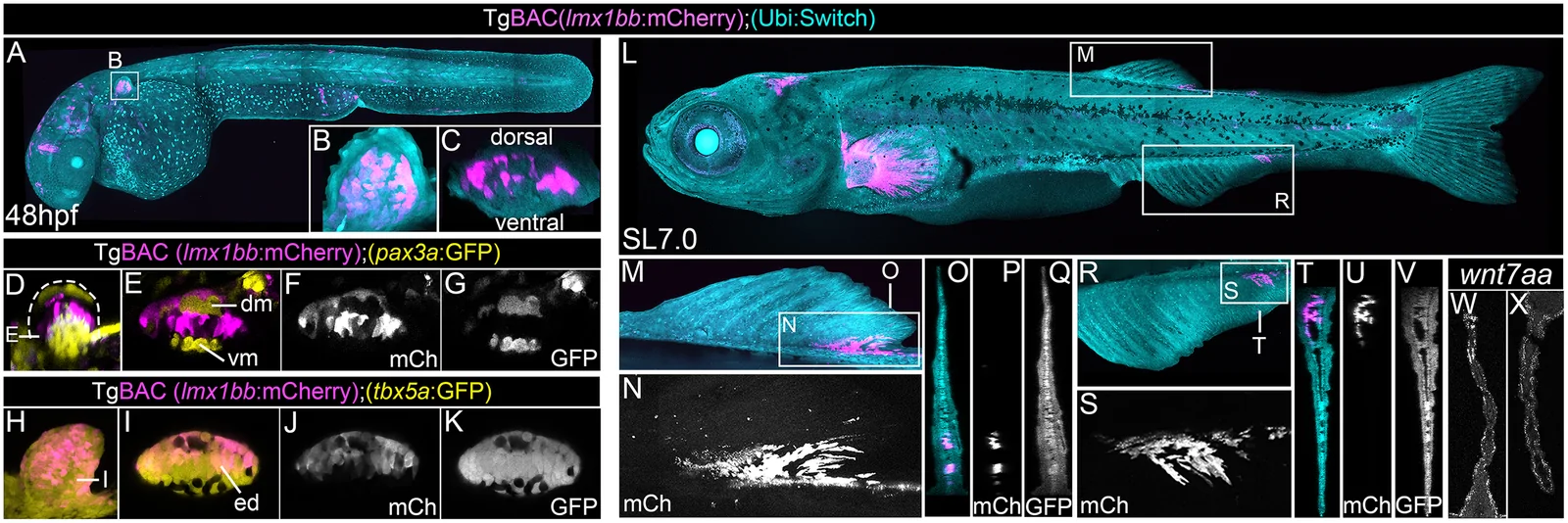
TgBAC(*lmx1bb*:mCherry) transgenic line reveals regionalized *lmx1bb* expression in both paired and median fins of zebrafish. TgBAC(*lmx1bb*: KalTA4_mCherry)^CH211-81G2^; (UAS-Elb:Eco-nfsb-mCherry) [hereafter referred to as TgBAC(*lmx1bb*:mCherry)]; (*ubi*:Switch) zebrafish at 48hpf. **(A–C)** Whole embryo (A) and the pectoral fin bud (B) are shown in lateral view. Rendered section of the pectoral fin bud is shown in (C). **(D–G)** Pectoral fin bud of TgBAC(*lmx1bb*:mCherry);(*pax3a*:GFP) at 48 hpf shown in lateral view (D) and in rendered section (E–G). *lmx1bb*:mCherry fluorescence does not overlap the somitic myoblast marker *pax3a*:GFP. **(H–K)** Pectoral fin bud of TgBAC(*lmx1bb*:mCherry); (*tbx5a*:GFP) at 48 hpf shown in lateral view (H) and in rendered section (I–K). *lmx1bb*:mCherry fluorescence co-localizes with *tbx5a*:GFP in fin lateral plate mesoderm. **(L–V)** TgBAC(*lmx1bb*:mCherry)]; (*ubi*:Switch) zebrafish larvae at standard length (SL) 7.0 mm. Whole larvae (L), dorsal fin (M, N), and anal fin (R, S) in lateral view. Rendered sections through the dorsal fin (O–Q) and anal fin (T–V). *lmx1bb*:mCherry fluorescence labels the posterior dorsal and anal fin mesenchyme. **(W, X)** HCR fluorescent in situ hybridization for *wnt7aa* on sections through the dorsal fin (W) and anal fin (X) reveals ectodermal labeling. Abbreviations: dm, dorsal muscle mass; GFP, green fluorescent protein; mCh, mCherry; vm, ventral muscle mass.

Given that median fins lack a patterning axis topologically equivalent to the DV axis of limbs, we next characterized BAC(*lmx1bb*:mCherry) expression during median fin development. In zebrafish, two distinct median fins—the dorsal fin and the anal fin—develop from condensations that emerge along a continuous median fin-fold [42,84–87]. Strikingly, regionalized BAC-*lmx1bb* fluorescence was present in SL 7.0 mm larvae at the posterior margin of both the dorsal and anal fins (Fig 1L–1V). Rendered cross-sections of the BAC(*lmx1bb*:mCherry);(*ubi*:Switch) line confirmed mCherry positive cells were specific to the fin-fold mesenchyme in both median fin types (Fig 1O–1Q, 1T–1V). Given the regionalized expression of *lmx1bb* in median fins, we next sought to determine if other core DV patterning genes were expressed during zebrafish median fin development. Using HCR fluorescent in situ hybridization (FISH), we detected expression of *wnt7aa* along the length of the ectoderm of both the dorsal and anal fins, as well as regions of trunk ectoderm more broadly, but failed to detect *en1* paralogues (Fig 1W, 1X)*.* Together, these results show that zebrafish dorsal and anal fins exhibit the same spatial neighboring of ectodermal *wnt7aa* and adjacent mesenchymal *lmx1bb* expression posteriorly that characterizes the dorsal compartment of paired appendages.

### *Wnt7a-Lmx1b* expression in median fins is a conserved feature of jawed vertebrates

To determine if the expression of “DV” patterning genes in median fins is a broadly conserved feature of actinopterygians, we next examined the expression of *lmx1b, wnt7a*, and *en1* orthologues in the paddlefish *Polyodon spathula* (Stages 41 and 43; during fin-fold elaboration) (Fig 2A–2C) as a representative basal (nonteleost) actinopterygian. In both the pectoral and pelvic fins, *wnt7a* and *en1* transcripts were detected in the dorsal and ventral ectoderm, respectively. *lmx1b* expression was present in the dorsal mesenchyme of both fin pairs, but in the pelvic fins it was posteriorly restricted. Like zebrafish, paddlefish possess single dorsal and anal fins [88]. In these median fins, *lmx1b* expression was restricted to the posterior fin mesenchyme and *wnt7a* was expressed more broadly across the fin ectoderm, whereas *en1* was absent.

**Fig 2:**
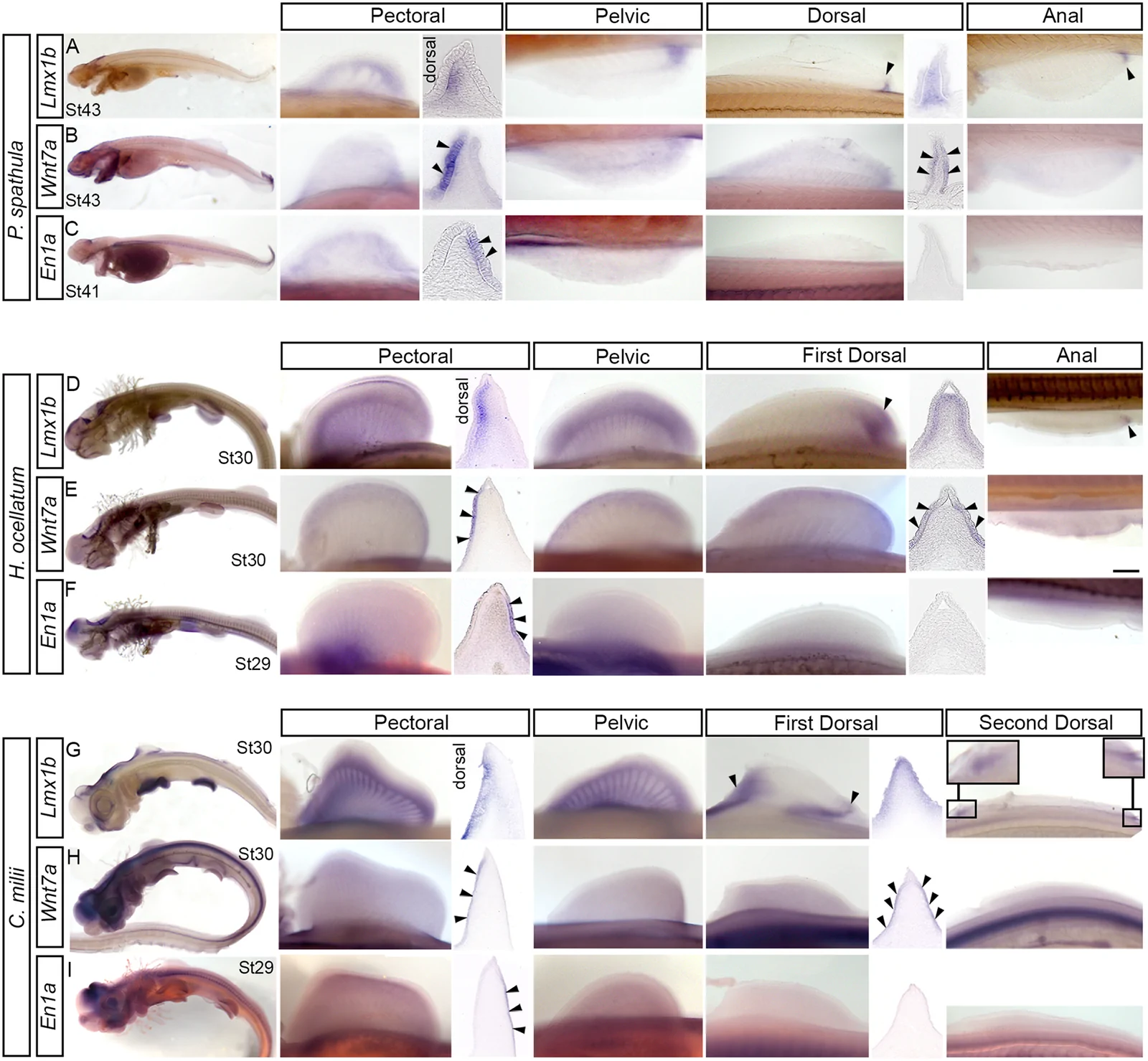
Comparative expression of dorsoventral patterning genes in paired and median fins. *lmx1b, en1,* and *wnt7a* expression for the paddlefish *Polyodon spathula* **(A–C)**, epaulette shark *Hemiscyllium ocellatum* **(D–F)**, and elephant fish *Callorhinchus milii* **(G–I)**. Whole embryos are shown in lateral view, anterior is left. Whole pectoral and pelvic fins are shown in ventral view, anterior is left. Dorsal and anal fins are in lateral view, anterior is left. For all cross-sections, dorsal is left, and ventral is right. Arrowheads in sections highlight ectodermal labeling. Arrowheads in whole mounts highlight regionalized expression of *lmx1b*.

To further broaden our comparative framework, we next investigated the expression of “DV” patterning genes in the paired and median fins of epaulette shark (*Hemiscyllium ocellatum*) and elephant fish (*Callorhinchus milii*), representatives of the two major extant lineages of cartilaginous fishes: elasmobranchs (Fig 2D–2F) and holocephalans (Fig 2G–2I), respectively. Both taxa are basal in the gnathostome phylogeny, with *C. milii* (family *Callorhinchidae*) considered to be the most basal of extant holocephalans [89], making it a critical species for examining hypotheses on the origin of paired appendages. In both taxa, the pectoral and pelvic fins exhibited broad expression of *wnt7a* in the dorsal ectoderm, *en1* in the ventral ectoderm, and *lmx1b* in the dorsal mesenchyme. Epaulette sharks possess two morphologically similar dorsal fins and an anal fin. In these unpaired fins, *lmx1b* staining was restricted to the posterior mesenchyme and *wnt7a* was expressed throughout the ectoderm, whereas no expression of *en1* was detected, consistent with the patterns observed in zebrafish (Figs 1 and S2) and paddlefish (Fig 2A–2C). Unlike epaulette sharks, the two dorsal fins of elephant fish differ morphologically. The first dorsal fin is characterized by a mineralized spine and a sail-like sheet, and the second forms as an elongate fin along the tail [90,91]. Whereas *wnt7a* was expressed along the length of both dorsal fins, *lmx1b* expression was detected in the mesenchyme of both the anterior and posterior domains of each dorsal fin. Collectively, these findings suggest the topological arrangement of *wnt7a*-positive ectoderm adjacent to *lmx1b*-positive mesenchyme in median fins is a shared feature of jawed vertebrates and was likely established prior to the origin of paired fins [76]. Furthermore, the spatial profile of median fin and pelvic fin *lmx1b* expression can vary between lineages, indicating evolutionary flexibility in how *lmx1b*-mediated networks are deployed.

### Regionalized contributions of the lmx1bb lineage differ between the paired and median fins but include common cell fates

Although *lmx1b* is expressed in mesenchymal cells during both paired and median fin development, it is unknown whether *lmx1b*-expressing cells are maintained throughout fin morphogenesis, which lineages they contribute to, and whether their spatial distribution differs between fin types. To compare *lmx1bb*-expressing progenitor contributions and fates between paired and unpaired fins, we performed a genetic lineage analysis by crossing the BAC(*lmx1bb*:mCherry-KalTA4) line with a Tg(UAS:iCre); (*ubi*:Switch) compound transgenic line. In resulting progeny, cells with a history of *lmx1bb* BAC-mediated iCre activation undergo a genetic recombination at the *ubi*:Switch locus, resulting in a change from GFP to mCherry fluorescence [92]. Triple transgenic fish were raised to early juvenile stages (SL8–12 mm), at which point the skeletal pattern of the pectoral, dorsal, and anal fins is largely established [87,93,94]. Tg(UAS:iCre);(*ubi*:Switch) fish were used as a negative-control (Fig 3).

**Fig 3:**
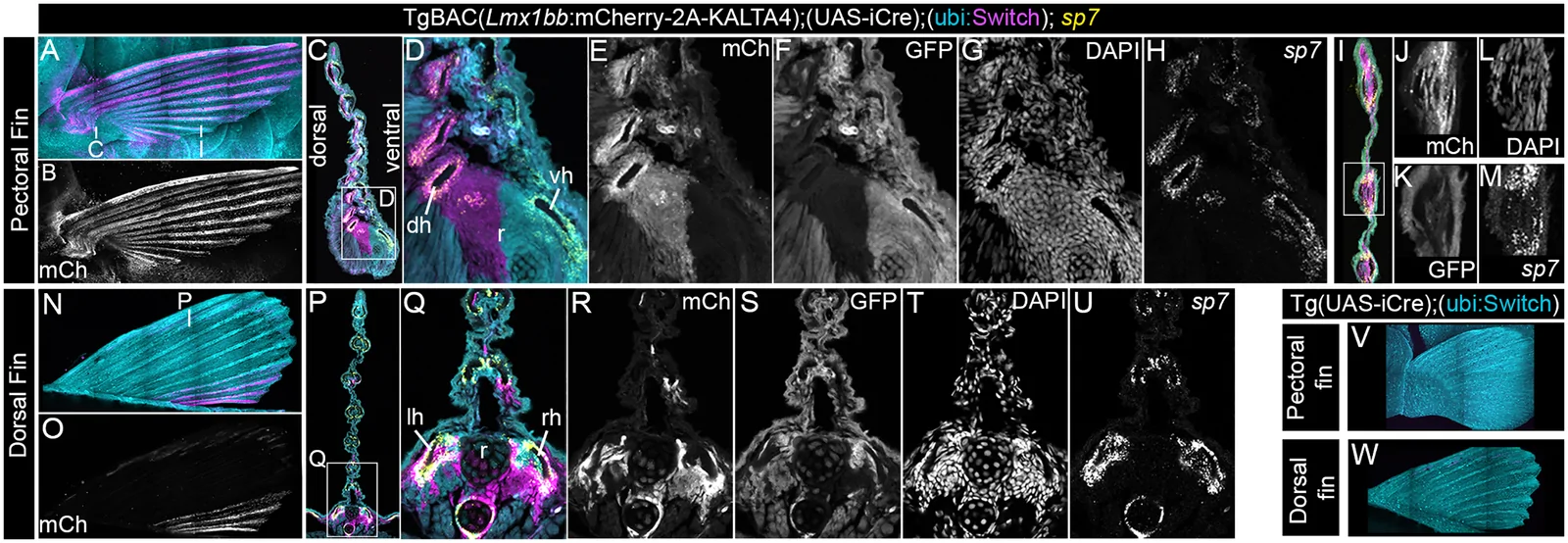
lmx1bb genetic lineage tracing in zebrafish pectoral and dorsal fins. Cre-mediated genetic lineage tracing using TgBAC(*lmx1bb*:mCherry-2A-KALTA4);(UAS-iCre);(ubi:Switch). Cells with a history of BAC-*lmx1bb* activation express mCherry, whereas all other cells ubiquitously express GFP. **(A, B)** Whole pectoral fin of zebrafish (standard length, SL = 11 mm) in lateral view; anterior is left. **(C–M)** Cross-sections through the proximal **(C–H)** and distal **(I–M)** pectoral fin. Merged channels are shown in **(C, D, I).** Single channels show mCherry **(E, J)**, GFP **(F, K)**, and DAPI **(G, L)**. HCR fluorescent in situ hybridization for the osteoblast marker *sp7* is shown in **(H, M)**. **(N, O)** Whole dorsal fin of zebrafish (SL = 11 mm) in lateral view; anterior is left. **(P–U)** Cross-section near the posterior margin of the dorsal fin. Merged channels are shown in **(P, Q)**. Single channels show mCherry **(R),** GFP **(S)**, and DAPI **(T)**. HCR fluorescent in situ hybridization for *sp7* is shown in **(U)**. **(V, W)** Pectoral **(V)** and dorsal **(W)** fins of negative-control Tg;(UAS-iCre);(ubi:Switch) zebrafish (SL = 8 mm) in lateral view; anterior is left. Abbreviation: mCh, mCherry.

In the pectoral fins of BAC(*lmx1bb*:mCherry-KalTA4);(UAS:iCre);(*ubi*:Switch) fish, mCherry labeled cells were visible in the endoskeletal radials and interspersed between myofibres of the adductor muscles (Figs 3A–3G and S3), consistent with their lateral plate derivation. Contributions to the pectoral fin rays (i.e., dermal skeleton) varied along the length of the fin. Proximally, mCherry positive cells surrounded the dorsal half of the fin rays, the dorsal hemitrichia, including along their articulation with the fin endoskeleton (Fig 3C–3G). In contrast, in the distal portion of the rays, mCherry positive cells lined both the dorsal and ventral hemitrichia (Fig 3I–3K, 3M), demonstrating a loss of fidelity of *lmx1bb*-positive cells to the dorsal fin compartment. Given observations that osteoblasts line the hemitrichia of caudal fins [95], we next sought to determine the identity of cells of the *lmx1bb* lineage positioned in close association with the dorsal hemitrichia of pectoral fins. FISH for the osteoblast marker *sp7* confirmed *lmx1bb* patterned cells give rise to osteoblasts of the dorsal but not ventral hemitrichia proximally (Fig 3D, 3E, 3H), but form both dorsally and ventrally positioned osteoblasts distally (Fig 3I–3M).

In dorsal fins, BAC(*lmx1bb*:mCherry-KALTA4) driven iCre resulted in mCherry positive cells in the posterior region of the fin, contributing to the dorsal fin radials as well as to both the left and right hemitrichia of the last several rays (Fig 3N–3T). Similar to the pectoral fins, FISH for *sp7* confirmed that cells of the *lmx1bb* lineage differentiated into osteoblasts of the hemitrichia near their articulation with the endoskeleton (Fig 3Q, 3R, 3U); however, unlike paired fins, this labeling was symmetric across the midline in the unpaired fins. Collectively, these results demonstrate that although the *lmx1bb* lineage contributes to different regions of paired versus median fins, the skeletal derivatives of the *lmx1bb* lineage are similar.

### *LARM cis*-regulatory modules are required for zebrafish *lmx1bb* expression in paired but not median fins

The similar topologies of ectodermal *wnt7a* and mesenchymal *lmx1b* expression in both paired and median fins, together with common cell fates derived from the *lmx1bb* lineage, suggest that a pre-existing “DV” appendage regulatory module may have been co-opted from the midline to the flank with the emergence of paired fins. However, the distinct, regionalized domains of *lmx1bb* expression between fin types imply divergent regulatory mechanisms for *lmx1b* induction. In mice, two *cis-*regulatory modules, *LARM1* and *LARM2,* drive limb *lmx1b* expression, and their loss leads to near-complete abrogation of *lmx1b* transcription and a bi-ventralized phenotype comparable to that of *lmx1b* null mutants. To understand how the *lmx1b* enhancer landscape evolved in vertebrates, we first generated VISTA sequence alignments [96] of regions flanking the *lmx1b* locus (Fig 4A) using a spotted gar viewpoint [97]. Although *LARM1* and *LARM2* have been previously proposed as unique to lobe-finned fishes [61], we identified conserved *LARM1* peaks in representative ray-finned and cartilaginous fishes, and *LARM2* peaks in ray-finned fishes, suggesting at least a subset of the *LARM* regulatory landscape is conserved across jawed vertebrates. In zebrafish, conserved *LARM1* and *LARM2* sequence elements were positioned ~19.5 and 24.3kb upstream of the lmx1bb transcription start site, respectively, in the intergenic region between *lmx1bb* and the neighboring gene *mvb12bb* (S1A Fig). Notably, *LARM1* and *LARM2* sequence elements were included in the BAC promoter fragment used to make the BAC(*lmx1bb*:mCherry) transgenic line.

**Fig 4:**
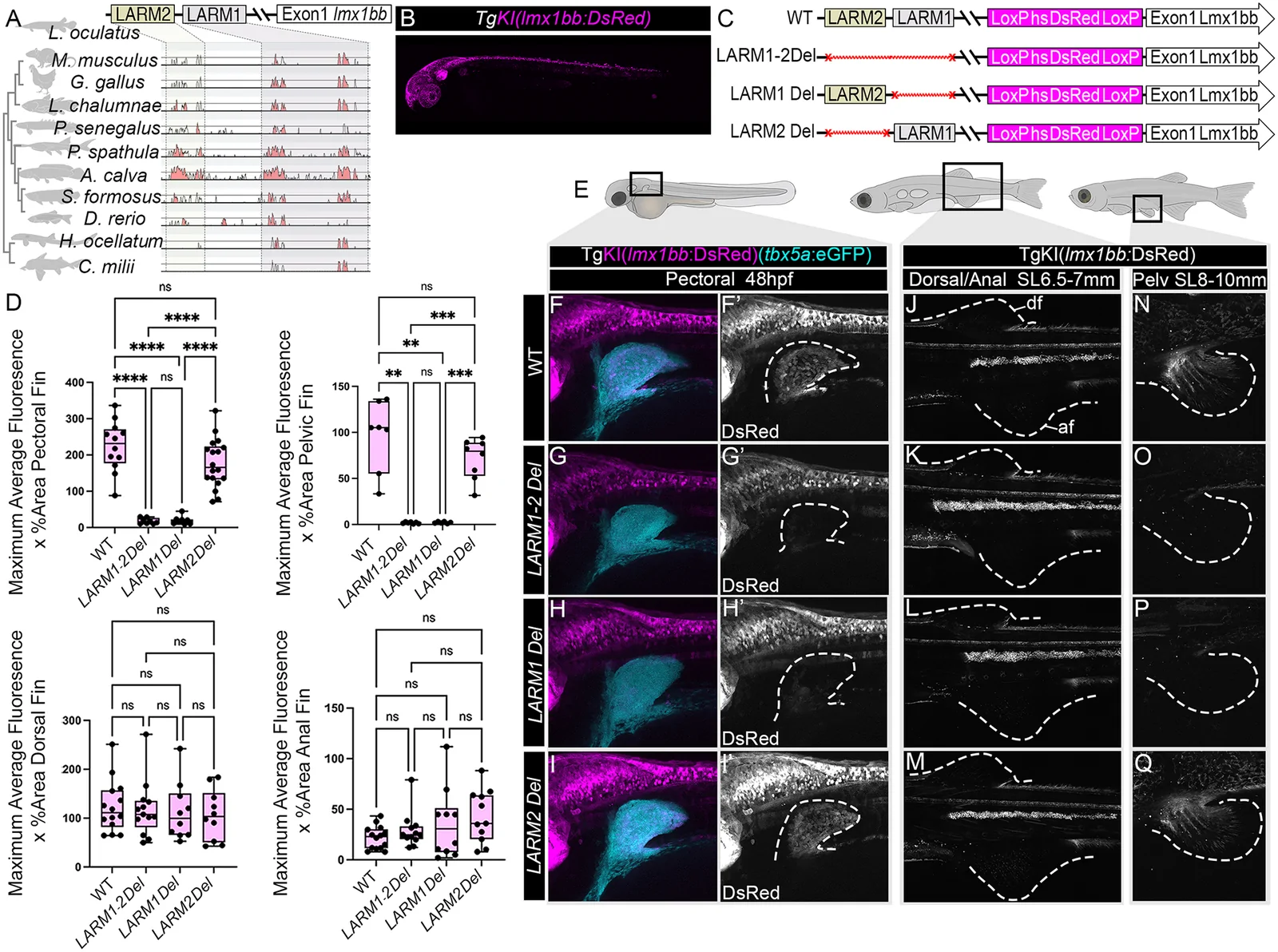
The *LARM1 cis*-regulatory module is required for paired but not median fin *lmx1bb* expression. **(A)** VISTA LAGAN global pairwise sequence alignments of the LARM locus for mouse (*Mus musculus*), chick (*Gallus gallus*), coelacanth (*Latimeria chalumnae*), Senegal bichir (*Polypterus senegalus*), paddlefish (*Polyodon spathula*), bowfin (*Amia calva*), Asian bonytongue (*Scleropages formosus*), zebrafish (*Danio rerio*), epaulette shark (*Hemiscyllium ocellatum*), and elephant fish (*Callorhinchus milii*) with a spotted gar (*Lepisosteus oculatus*) viewpoint. *LARM1* is partially conserved among jawed vertebrates and *LARM2* among bony fishes. **(B)** Tg(*lmx1bb*-LoxP-hsDsRed-LoxP-DTA) knock-in line [referred to hereafter as TgKI(*lmx1bb*:DsRed)] at 48 hpf in lateral view, anterior is left. **(C)** *LARM* deletion series schematic. Single deletions of *LARM1, LARM2*, or a region bracketing *LARM1* + *LARM2* were generated in cis to the TgKI(*lmx1bb*:DsRed) allele. The red line indicates the deleted fragment. **(D–Q)** Average maximum fluorescence was compared between WT and *LARM* deletions in cis to the TgKI(*lmx1bb*:DsRed) allele in pectoral (D, F–I’), dorsal, and anal (D, J–M), and pelvic (D, N –Q) fins. All fins are shown in lateral view, anterior is left. The BAC(*tbx5a*:eGFP) transgene is used to visualize the position of the pectoral fin (F–I). In single-channel images for DsRed, dashed lines mark fin margins (F’–I’). *LARM1-LARM2* deletions and single *LARM1* deletions resulted in reduced dsRed fluorescence relative to WT for paired but not median fins. Statistical analyses in (D) for pectoral, pelvic and anal fins were done using Welch’s one way ANOVA with Dunnett’s T3 post hoc multiple-comparisons test; and for dorsal fins using a one-way ANOVA with Tukey’s multiple-comparisons test. Underlying data are provided in S1 Data. ns is not significant, * is *p* < 0.05, ** is *p* < 0.01, *** is *p* < 0.001, and **** is *p* < 0.0001.

In order to test the function of *LARM* enhancers in fins, we used CRISPR-Cas9 mediated mutagenesis to generate a series of single-allele germline deletions encompassing regions of sequence conservation for *LARM1*, *LARM2*, or both *LARM1* plus *LARM2* in cis with a *lmx1bb* knock-in reporter allele [Tg(*lmx1bb*-LoxP-hsDsRed-LoxP-DTA), hereafter referred to as TgKI(*lmx1bb*:DsRed)] (Figs 4B, 4C and S4A). We then compared fin-specific maximum average fluorescence across mutant and wild type *LARM* backgrounds, providing a direct readout of enhancer function. In both pectoral (48 hpf) (Fig 4D–4I’ and S1 Data) and pelvic fin buds (SL 8–10 mm) (Fig 4D, 4N–4Q and S1 Data), complete deletion of *LARM1*–*LARM2* caused a dramatic reduction in DsRed fluorescence (*p* < 0.0001 for pectoral fins; *p* = 0.0032 for pelvic fins). A similar reduction of reporter fluorescence was observed following the singular deletion of *LARM1* (*p* < 0.0001 for pectoral fins; *p* = 0.0033 for pelvic fins), whereas no significant reduction in fluorescence was observed following the deletion of the region encompassing *LARM2*. These results demonstrate that *LARM* enhancer activity in paired appendages was already established in the last common ancestor of bony fishes. Furthermore, *LARM* regulatory modules function during the development of both pectoral and pelvic fins, with *LARM1* a principal, paired fin enhancer in zebrafish.

We next sought to test if *LARM* enhancers are required for *lmx1bb* expression in developing dorsal and anal fins, as might be predicted by models of median fin regulatory network co-option during the origin of paired fins. In contrast to pectoral and pelvic fins, complete deletions of *LARM1–LARM2* or single deletions of *LARM1* or *LARM2* did not significantly affect dsRed fluorescence in the dorsal or anal fins (Fig 4D, 4J–4M and S1 Data). Together, these results provide evidence that distinct *LARM-*dependent and independent regulatory systems operate in zebrafish paired versus median fins, respectively.

Given the role of *wnt7a* as an upstream regulator of *lmx1b* in limbs, and the expression of *wnt7aa* in the pectoral [70], dorsal, and anal fins of zebrafish larvae (Fig 1W, 1X), we generated stable *wnt7aa* and *wnt7ab* mutant lines with a premature stop in the second and third exons, respectively (S5A and S5B Fig), both of which are predicted to cause loss of function. These lines were subsequently crossed to generate *wnt7aa;wnt7ab* double mutants, which were then back-crossed with our TgKI(*lmx1bb*:DsRed) knock-in allele. No differences in *lmx1b* driven DsRed fluorescence were observed in the dorsal fins of WT fish compared to all allelic combinations of *wnt7aa*;*wnt7ab* mutants, indicating neither wnt7a paralogue is required for dorsal fin *lmx1bb* expression in zebrafish (S5C and S5D Fig and S1 Data).

### Deletion of *LARM* enhancers affects zebrafish paired fin but not median fin patterning

To determine if *LARM* enhancers are selectively required for patterning of paired versus median fins in zebrafish, we generated a germline *LARM1–LARM2* deletion mutant in a WT background (S4B Fig). Progeny of in-crossed heterozygotes were raised to late sub-adult stages (~1.7–2.4 cm SL), scanned by micro-computed tomography (µCT), and digital reconstructions were used to assess skeletal pattern. In the pectoral fins of wild type (*n* = 4) and heterozygous (*n* = 8) zebrafish, the basal portion of the dorsal and ventral hemitrichia were asymmetric across the midline, such that the dorsal hemitrichia formed a relatively smooth border near their articulation with the endoskeleton, whereas the ventral hemitrichia formed a prominent saw-tooth pattern when visualized in lateral and medial views (Fig 5A–5D). Strikingly, in all *LARM1–LARM2* null mutants scanned (*n* = 5), the pattern of the ventral hemitrichia was duplicated in the dorsal fin compartment, resulting in a bi-ventralized phenotype (Fig 5E–5H).

**Fig 5:**
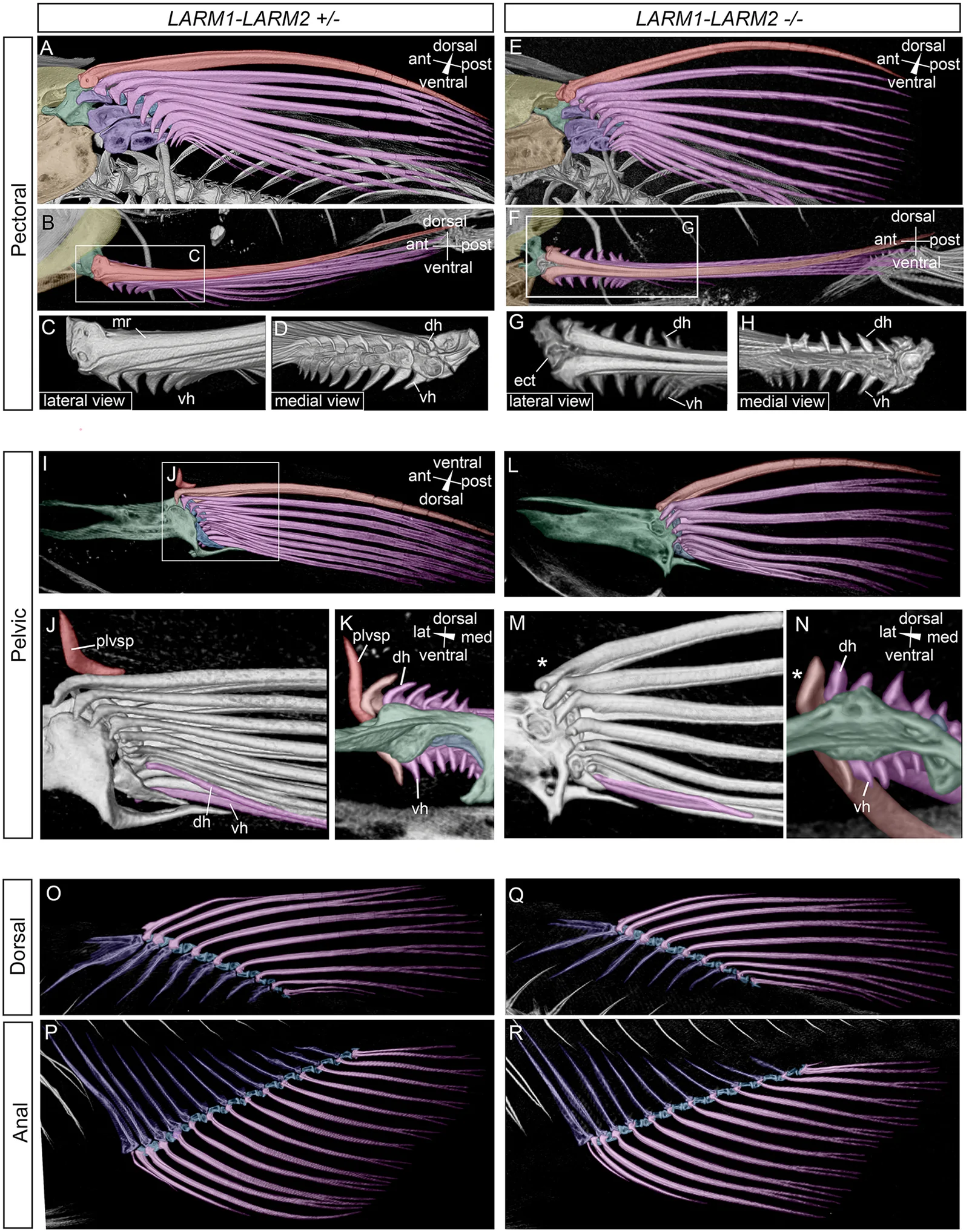
*LARM* deletion results in aberrant skeletal patterning of the paired fins but not median fins. Digital renderings of the fin skeleton from microcomputed tomography scans. **(A–H)** Pectoral fins of *LARM1–LARM2*^+/–^ heterozygotes (A–D) and *LARM1-LARM2*^–/–^ homozygous (E–H) mutants. In (A, B, E, F), the skeletal elements of the shoulder girdle and pectoral fin are pseudocolored as follows: cleithrum, yellow; coracoid, brown; scapula, green; proximal radials, blue; distal radials, cyan; marginal ray, pink and all other rays magenta. Radials are partially removed in (C), (D), (G), and (H). The dorsal hemitrichia (dh) of the fin rays appear bi-ventralized in *LARM1–LARM2*^–/–^ mutants and form a sawtooth pattern similar to the ventral hemitrichia (vh). (**I–N)** Pelvic fins of *LARM1-LARM2*^+/–^ heterozygotes (I–K) and *LARM1-LARM2*^–/–^ homozygous (L–N) mutants. In (I, K, L, and N), the pelvic girdle is pseudocolored green; radials, blue; the marginal ray, pink; and all other rays magenta. In (J), a single ray is pseudocolored magenta to highlight lateral curvature of the dorsal hemitrichia, which forms a “Y” shape with the ventral hemitrichia in *LARM1-LARM2*^+/–^ heterozygotes. In *LARM1-LARM2*^–/–^ mutants, this curvature is lost, the dorsal hemitrichia appear ventralized, and the pelvic splint (plvsp, red) is absent (*). **O–R**. No differences were observed in the dorsal (O, Q) or anal (P, R) fins between LARM1-LARM2^+/–^ heterozygotes (O, P) and LARM1-LARM2^–/–^ homozygous (Q, R) mutants. The proximal radials are pseudocolored blue; the distal radials, cyan; and the fin rays, magenta. ect, ectopic bone; mr, marginal ray.

Like the pectoral fins, loss of *LARM1–LARM2* also resulted in dorsal duplications of ventral morphologies in the pelvic fins. In wild type and heterozygous fish, the dorsal hemitrichia curve laterally after splitting from the ventral hemitrichia, creating a deep Y-shape that is most pronounced for the medial rays (Figs 5I, 5J, S6A and S6B). In contrast, in homozygous mutants, the dorsal hemitrichia remain in register with the ventral hemitrichia, resulting in a narrower “Y” (Figs 5L, 5M, and S6C–S6E, *p* = 0.0017) (S1 Data). Furthermore, the shape of the proximal ends of the dorsal hemitrichia appears ventralized in homozygous mutants relative to heterozygotes (Fig 5N). We also identified a prominent, dorsally directed spine lateral to the first fin ray in all wild type (*n* = 4) and a subset of heterozygous (50%, *n* = 4/8) fish examined by microCT. To the best of our knowledge, this element has not been reported in zebrafish but appears similar to the pelvic splint reported in a variety of teleost fishes [98–100]. This bone was absent in all homozygous mutants (*n* = 0/5), indicating its formation is *LARM-*enhancer dependent (S6F–S6J Fig, *p* = 0.0079) (S1 Data).

In addition to the paired fins, we examined the dorsal and anal fins of *LARM1/LARM2*^+/+^, *LARM1/LARM2*^+/–^, and *LARM1/LARM2*^–/–^ fish. We did not observe any difference in the number of rays or skeletal morphologies (Fig 5O–5R). Collectively, these data are consistent with our enhancer-deletion knock-in reporter assays (Fig 4) and demonstrate that the *LARM* regulatory module is essential for dorsoventral patterning of the paired fin dermal skeleton but is not required for patterning of the median fins in zebrafish.

Because *LARM1-LARM2* deletion removes most but not all reporter expression (Fig 4), we sought to compare enhancer deletion phenotypes with those of *lmx1bb* null mutants. Using CRISPR-Cas9 gene editing, we generated a stable germ line *lmx1bb* mutant with a premature stop in the third exon (S7A Fig). However, homozygous mutants were embryonic lethal, precluding analyses of skeletal phenotypes (S7B Fig).

## Discussion

The evolutionary acquisition of paired fins created a new dorsoventral anatomical axis. Defining the cellular and molecular bases for establishing dorsal versus ventral morphologies is therefore central to understanding early fin evolution. We have mapped the contributions of the *lmx1bb* lineage to mature pectoral and dorsal fin anatomy in zebrafish, revealing common chondrocyte, connective tissue, and dermal skeleton osteoblast fates across fin types. These findings broadly align with *lmx1b* Cre-based lineage tracings in mouse limbs, which similarly identified chondrocyte and connective tissue derivatives [58,101]. Fin ray reduction and eventual loss in the tetrapod lineage would have required loss of a dermal skeletal fate from the *lmx1b*-lineage tree, potentially occurring through the erosion of direct Lmx1b-responsive *cis-*regulatory targets. However, because fin rays persist in *LARM* deletion mutants, it is unlikely that loss of Lmx1b-mediated pathways would have been a primary driver of dermal skeleton loss. Instead, such changes were likely integrated within a broader suite of developmental mechanisms for dermal skeletal reduction and endoskeletal elaboration [102–109].

In zebrafish, deletion of *LARM* regulatory modules causes a deficit in *lmx1b* expression in the pectoral and pelvic fins and results in fin ray patterning defects. Strikingly, the bi-ventralized phenotype of the pectoral fin dorsal hemitrichia closely correlates with the distribution of *lmx1b* lineage-derived osteoblasts. A recent bioRxiv preprint by Hawkins and colleagues [110] (now published [111]) similarly used zebrafish mutants to dissect *LARM* enhancer function during paired and median fin development and, through cross-species transgenic assays, showed that *LARM* elements from diverse gnathostomes, including skate and catshark, can drive fluorescent reporter expression in zebrafish pectoral fins. Our *LARM* deletion phenotypes broadly agree with those of Hawkins and colleagues [110,111], together supporting deep conservation of an *lmx1b* dorsal determination program in paired fins that is regulated by a *LARM cis-*regulatory module.

Interestingly, Hawkins and colleagues [110,111] also reported that whereas limb-like Wnt7a-Engrailed-Lmx1b epistasis is maintained in early zebrafish pectoral fins, *en1a; en1b* double mutant zebrafish do not exhibit an overt pectoral fin phenotype, as would be predicted with ventral expansion of *lmx1b* expression [48,112]. In mice, the bi-dorsalized phenotype of *En1* mutants predominantly affects the autopod (i.e., distal-most region of limb) [48,113,114]. Our Cre-mediated fate mapping in zebrafish shows that dorsal-specific contributions of *lmx1bb*-expressing cells are not maintained in distal fin rays where both dorsal and ventral hemitrichia derive from a *lmx1bb*-positive lineage. Given evidence for generative homologies between the autopod and fin rays [102], this loss of dorsal lineage fidelity may help explain the absence of an *en1* mutant phenotype in fins.

Comparative expression analyses support the conservation of ectodermal *wnt7a* and mesenchymal *lmx1b* expression domains in the unpaired fins of gnathostomes [76,110,111]. Although shared expression patterns can suggest regulatory network co-option, such similarities can also arise convergently. Demonstrating that the same *cis-*regulatory element drives expression of a network component in two different developmental contexts provides a key test of co-option models [115]. The long-range ZRS enhancer for *Shh* is one such example, functioning in both limbs and unpaired fins [41]. In zebrafish, however, deletion of the *LARM cis-*regulatory module affects pectoral and pelvic fins but not median fins (see also Hawkins and colleagues [110,111]). Similarly, *wnt7a* ligands are dispensable for dorsal fin *lmx1bb* expression, an observation that aligns with pharmacological manipulation experiments in the cichlid *Astatotilapia burtoni* [76]. Collectively, these observations run counter to predictions of simple gene network co-option [110,111].

How a median fin *lmx1b* expression domain was effectively “rotated” 90^o^ in paired fins and integrated with flank-derived regulatory inputs is not well understood, but likely occurred in the context of a primitive body wall ectoderm already compartmentalized along the DV axis by *engrailed* [74,76,116]. The paired-fin specificity of the *LARM*-deletion phenotype, together with the conserved function of *LARM1* in zebrafish and mouse, suggests that the acquisition of *LARM* enhancers may have been an important early step in the assembly of the dorsoventral patterning system of paired appendages (see also refs [110,111]). Under this model, the activation of *lmx1b* in flank lateral plate through novel enhancer activity may have facilitated recruitment of a broader *lmx1b-*mediated network already established in median fins [76]. Given the diverse developmental pathways regulated by Lmx1b in limbs [62], deployment of this network, at least in part, could have driven dorsoventral asymmetries in patterning of the skeleton and musculature, ECM organization, and motor axon pathfinding (see also ref [76]). Functional genomic studies defining *lmx1b* regulation and downstream network architecture in the paired and unpaired fins of diverse taxa, including representative chondrichthyans, will provide a critical test of this model. Collectively, the findings described here address a key limitation of simple co-option models for the origin of paired fins and inform a new molecular framework for understanding how a dorsoventral axis, absent in median fins, may have evolved in paired appendages.

## Methods

### Animal handling and transgenic lines

Adult zebrafish were maintained in a recirculating freshwater aquarium system kept at approximately 28 °C with a 14-hour light, 10-hour dark cycle using standard husbandry procedures [117]. Zebrafish work was done in accordance with Monash Animal Ethics Committee approved project IDs 22161, 25827, 41803 and 47944. Previously described lines included TgBAC(*pax3*:GFP)^i150^ [82], Tg (*tbx5a*:eGFP) [79,80]; and Tg(*ubi*:Switch) [92]. The Tg(*lmx1bb*-LoxP-hsDsRed-LoxP-DTA) line was obtained from the National BioResource Project, Zebrafish (https://shigen.nig.ac.jp/zebra/). Embryos of the paddlefish *Polyodon spathula* were obtained from Osage Catfisheries (Osage Beach, MO, USA) and raised in 18 °C freshwater tanks at Kennesaw State University (Kennesaw, GA, USA) as previously described [103] and in accordance with KSU Institutional Animal Care and Use Committee approved protocols 12-001, 16-001. Embryos of the Epaulette shark *Hemiscyllium ocellatum* were obtained from an adult brood stock maintained at Monash University (Clayton, VIC, Australia), using procedures for Epaulette husbandry, breeding, and egg collection as previously described [118] and in accordance with protocols approved under Monash University Animal Ethics Project IDs 30347 and 44673. Embryos of the Elephant fish *Callorhincus milii* were obtained from wild-caught adults temporarily maintained in captivity for egg laying using previously described capture and husbandry protocols [119] in accordance with Monash University Animal Ethics Project IDs 22254 and 40651. Staging of Paddlefish, Epaulette shark, and Elephant fish was based on references [88,90,120,121].

To generate the TgBAC(*lmx1bb*: KalTA4_mCherry)^CH211-81G2^ strain, the CH211-81G2 clone was obtained from the BACPAC resource center (https://bacpacresources.org) and modified by the Monash Genome Modification platform to generate the final Lmx1bb BAC clone used for injection. In brief, a KalTA4-T2A-mCherry-polyA-FRT-neomycin-FRT cassette was inserted into the CH211-81G2 clone, deleting the coding sequence of the first exon of the *lmx1bb* gene. FLP recombination was used to remove the neomycin resistance cassette. Finally, an iTOL2 - KM cassette was added and the BAC simultaneously trimmed down to a total length of ~103 Kb. The final BAC was injected at 100 ng/μl into one-cell-stage wildtype embryos along with transposase RNA (25 ng/μl) synthesized from the pcs2FA-transposase vector using an mMessage machine Sp6 kit (Ambion, AM1340). Injected embryos were manually sorted for mCherry fluorescence. Positive embryos were raised to adults and outcrossed for founders to establish a stable line.

CRISPR/Cas9-mediated gene editing was used to generate mutants used in this study. crRNA sequences were designed using either the IDT (https://sg.idtdna.com/site/order/designtool/index/CRISPR_CUSTOM) or ChopChop (https://chopchop.cbu.uib.no) [122] online tools. *LARM1, LARM2,* and *LARM1-LARM2* mutants were generated by targeted deletion using the following pairs of crRNA sequences (PAM sequences in uppercase): *LARM1*: 5′-tgtagttaccggatgaaaccTGG-3′ and 5′-agcaccatagccccatgtccAGG-3′; *LARM2:* 5′-ggggctaagaagaacaggacAGG-3′ and 5′-tatgcctaactaactaagttAGG-3′**;** *LARM1-LARM2***:** 5′-tgtagttaccggatgaaaccTGG-3′ and 5′-tatgcctaactaactaagttAGG-3′. Targeting crRNAs were duplexed with tracrRNA according to the manufacturer’s protocol to generate gRNAs, which were subsequently injected along with Cas9 protein (Alt-R CRISPR–Cas9 system, IDT) into one-cell-stage heterozygous [Tg(*lmx1bb*-LoxP-hsDsRed-LoxP-DTA) embryos for LARM deletion mutants. Stable germline mutants for LARM enhancers in cis to the (*lmx1bb*-LoxP-hsDsRed-LoxP-DTA) transgene included a net deletion of 2682 bp for *LARM1*; a deletion of 764 bp for *LARM2*; and 6827 bp deletion for the region spanning *LARM1-LARM2*. The stable germline mutant for *LARM1-LARM2* deletion in a wild type background (used for assessing skeletal malformations) included a 6862 bp deletion. The *wnt7ab* mutant was generated using the following crRNA sequences (PAM sequences in uppercase): 5′-ggcttctatgctcatgacaaTGG-3′**,** 5′-tgcataacaatgaagtaggaCGG-3′, 5′-cgtctggagtgtaaatgtcaTGG-3′ resulting in the introduction of a premature STOP codon in exon three followed by a 8590 bp deletion extending into exon four. Mutants for *wnt7aa* and *lmx1bb* were generated by insertion of a premature STOP cassette at cut sites generated using the following crRNA sequences (PAM sequences in uppercase): *wnt7aa*: 5′-cgctcccaatgcaaccaccgAGG-3′; and *lmx1bb*: 5′-gacggagtgccatcaccaggCGG-3′. STOP cassette oligonucleotide design and co-injection was performed as previously described [123]. Oligonucleotide sequences included a universal STOP cassette and flanking gene-specific homology arms, and were as follows (STOP sequence underlined):

- *lmx1bb*: agggacggagtgccatcaccgtcatggcgtttaaaccttaattaagctgttgtagaggcggtgtgtgaggggtgc
- *wnt7aa*:
  actcgctcccaatgcaaccagtcatggcgtttaaaccttaattaagctgttgtagccgaggaaaagcccctgtaa

Primers used for genotyping of mutant lines are as follows: *LARM1* F aagctcttcaggtttgtatccg; *LARM1* R gttcagaaaagctttgtcgctt; *LARM2* F gttccagcacattaaggtctcc; *LARM2* R gctctgctcatactcaacaccac; *wnt7aa* F ttctccaatgacaatgatagcg; *wnt7aa* R ttctctcagtgcagcttggtta; *wnt7ab* F gcaatgtgcctcaacacaccttc; *wnt7ab* R gcacgtgttgcatttgacg; *wnt7ab* heterozygote R agccgactatcaagctggtg (to identify wild type alleles in heterozygotes); *lmx1bb* F cgtaatattattcggacgcctt; *lmx1bb* R: attagagcccattgcctgcg.

### cDNA synthesis, cloning and in situ hybridization

Cloning for ISH probe template was done as previously described [109]. RNA was isolated from pooled embryos with either Trizol reagent (Invitrogen) plus the PureLink Micro-to-Midi Total RNA Purification System (Invitrogen) for *P. spathula*, or with a RNAeasy Mini Kit (Qiagen) for *D. rerio, H. ocellatum, and C. milii,* and was used as template for synthesizing single-strand cDNA with the Superscript III First Strand Synthesis kit (Invitrogen), all per manufacturer’s instructions. Primer pairs for cloning probe fragments were as follows: *D. rerio wnt7aa* F atgagcaggaaaacgcg; *D. rerio wnt7aa* R ggttatacctctccttgaggatg; *D. rerio en1a* F atggaggatcagcggc; *D. rerio en1a* R tcgcttatcgtcgctttc; *D.rerio lmx1bb* F actttctcaagctgcagg; *D.rerio lmx1bb* R agctcctcatcttcgcttttc; *P. spathula Wnt7a* F gagaggactgtctttggaaaag; *P. spathula Wnt7a* R gtagcagcaccagaggaa; *P. spathula En1a* F atctgccttctcctctgatcctg; *P. spathula En1a* R gcacctgtctcagtccattcg*; P. spathula Lmx1b* F actctaggcgtgctgct; *P. spathula Lmx1b* R ccttgggtctttgccatcg*; H. ocellatum Wnt7a* F taggtggattctcttcggtgg; *H. ocellatum Wnt7a* R gtgttgtatcctcttccgcaaca; *H. ocellatum En1a* F gggagagcgtttctctctcg; *H. ocellatum En1a* R cgcttgtcctccttctcactcttc; *H. ocellatum Lmx1b* gctagacggcatcaaaatggag; *H. ocellatum Lmx1b* R gctttggtcgtctctgatcct; *C. milii Wnt7a F tctgcgtgggaatcgtttacc; C. milii Wnt7a R acaccaagtcagtccccatg; C. milii En1a F acgatcgcgtggagagtaga; C. milii En1a R gcttgtcctccttctcgctt; C. milii Lmx1b F gaaggaaaaggagctcctgag; C. milii Lmx1b R agcctgtcgatggggtta.*

PCR amplicons were cloned into pGEM-T Easy Vector (Promega) and verified by Sanger sequencing. For in situ hybridization, plasmids were linearized with restriction enzymes (New England Biolabs, NEB). Any 3′ overhangs were blunted using DNA Polymerase I, Large Klenow (NEB). In vitro transcription was carried out using SP6 or T7 RNA polymerases (Promega) and Digoxigenin RNA labeling mixes (Roche) according to the manufacturer's instructions. In situ hybridizations were as described in Modrell and colleagues [124]. Hybridization was carried out at 68–70 °C overnight. Digoxigenin-labeled probes were detected with Anti-Digoxigenin-AP, Fab fragments (1:2000 dilution; Roche) and developed with BM-Purple (Roche). Sense probe was used with stage-matched specimens as a negative-control.

### Hybridization chain reaction

Hybridization Chain Reaction RNA FISH probe against zebrafish *sp7* and *wnt7aa* were generated using a custom HCR Probe Designer [125] and included 27 probe pairs designed for use with the B3 amplifier for *sp7*; and 24 probe pairs designed for use with the B1 amplifier for *wnt7aa*. Probe sequences are presented in S1 Table. FISH was performed on slides according to manufacturer’s recommendations (https://www.molecularinstruments.com). Each probe set was used at a concentration of 0.4 pmol per 100 µL of hybridization buffer, and hybridization was carried out overnight at 37 °C in a humidity chamber. H1 and H2 hairpins were used at a concentration of 6 pmol per 100 µL of amplification buffer, and amplification conducted for ~18 hours at room temperature. Sections were imaged on a Zeiss LSM 980 Confocal Microscope.

### Fluorescence assays in TgKI(*lmx1bb*:DsRed) background

All wild type control and *LARM* deletion mutants in the TgKI(*lmx1bb*:DsRed) background, as well as *wnt7aa;wnt7ab* mutants in the TgKI(*lmx1bb*:DsRed) background, were imaged using a Zeiss 980LSM confocal microscope and Zen Blue edition software. Image acquisition settings were identical across wild type and mutant fish for each fin type. Image stacks were analyzed using Fiji (ImageJ) software. For LARM deletion analyses, a baseline minimum threshold for pixel intensity was set to 10 for pectoral and pelvic fins and to 20 for dorsal and anal fins. For *wnt7aa;wnt7ab* mutant analyzes, a baseline minimum threshold for pixel intensity was set to 10. Fins were traced in each Z-stack section, the mean intensity multiplied by % area of fluorescence within each fin tracing, and the maximum value used for each fish.

### VISTA alignments

Genome sequence data were obtained from Ensembl (https://asia.ensembl.org/index.html) or the NIH Genome database (https://www.ncbi.nlm.nih.gov/gdv). Conserved sequence elements were identified using mVISTA LAGAN (https://genome.lbl.gov/vista/mvista/submit.shtml) with translated anchoring to improve alignments of distal homologies, a 20 bp calc window, 40 bps min cons width, and a 70% conserved identity threshold.

### Micro-computed tomography

Zebrafish used for X-ray tomography were fixed overnight in 4% PFA, washed in PBS and mounted in plastic tubes in 1% agarose for scanning. Scans were carried out at the Monash Department of Civil Engineering on a Zeiss Xradia 520 Versa and image sets visualized using Avizo Software (Thermo Fischer Scientific).

### Statistical analysis

GraphPad Prism was used to analyze data sets. All graphs show the number of fish used in each experiment and underlying data are provided in S1 Data. For pectoral, pelvic, and anal fins, maximum average fluorescence between wild type, *LARM1-LARM2, LARM1*, and *LARM2* deletion mutants were compared using Welch’s one way ANOVA and Dunnett’s T3 multiple-comparisons test. For dorsal fins, assumptions of equal variances (Brown-Forsythe) were met, and one-way ANOVA was performed with Tukey’s multiple-comparisons test. Fin ray widths below the split of the dorsal and ventral hemitrichia were compared using a Kruskal–Wallis test with Dunn’s multiple-comparisons test (adjusted *p* values; α = 0.05). All other fin ray measurements were compared using a one-way ANOVA with Tukey’s multiple-comparisons test. *p* values less than 0.05 were considered significant.

## Supporting information

S1 Fig TgBAC(*lmx1bb:*mcherry) line recapitulates aspects of endogenous *lmx1bb* expression in zebrafish. **(A)** Position of CH211-81G2 BAC fragment used to generate the TgBAC(*lmx1bb:*mcherry) line. This BAC included regions of *lmx1bb* and *mvb12bb*. **(B)** TgBAC(*lmx1bb:*mcherry);(*ub*i:Switch) zebrafish at 48 hpf in lateral view. The ubi:Switch transgene labels all cells of the embryo. **(C)** In situ hybridization for *lmx1bb* in zebrafish embryo at comparable stage in lateral view.
(TIF)

S2 Fig Expression of *wnt7aa* and *en1a* in 48 hpf zebrafish. In situ hybridization for *en1a* **(A, B)** and *wnt7aa* **(C, D)** in 48hpf zebrafish embryos. Whole mounts are shown in lateral view, anterior is left (A, C). In section (B, D), dorsal is right, and ventral is left.
(TIF)

S3 Fig *Lmx1bb* lineage analysis in zebrafish pectoral fins. Genetic lineage analysis using the TgBAC(*lmx1bb*:mCherry-2A-KALTA4);(UAS-iCre);(ubi:Switch) transgenic line. Cells with a history of BAC-*lmx1bb* activation express mCherry (magenta), whereas all other cells ubiquitously express GFP (cyan). Actin staining (yellow) with phalloidin makes visible myofibres, and DAPI counterstain labels nuclei. **(A)** Cross-section through the proximal region of the pectoral fin of a zebrafish (Standard length, SL 11 mm). **(B–K)** Enlarged insets from (A) showing merged (B, G) and individual channels (C–F, H–K). mCherry positive cells contribute to fin radials (r), dorsal hemitrichia (dh), and surrounding connective tissues. Arrowheads (H) highlight subset of mCherry positive cells in adductor muscle (ad). Abbreviations: ab, abductor muscle; dh, dorsal hemitrichia; r, radials; vh, ventral hemitrichia.
(TIF)

S4 Fig *LARM* regulatory module deletion. **(A)** CRISPR/Cas9-mediated deletion of either *LARM1-2*, *LARM1*, or *LARM2* in cis with the knock-in reporter allele Tg(*lmx1bb*-LoxP-hsDsRed-LoxP-DTA). Sanger sequencing confirmed deletion of target sequence. **(B)** CRISPR/Cas9-mediated deletion of *LARM1–2* in wild type background. Sanger sequencing confirmed deletion of target sequence.
(TIF)

S5 Fig TgKI(*lmx1bb*:DsRed) fluorescence in the dorsal fin is not affected in *wnt7aa;wnt7ab* mutants. **(A, B)** CRISPR/Cas9-mediated introduction of a premature STOP codon (red asterisk) in the second exon of *wnt7aa* (A) and the third exon of *wnt7ab* (B). Pink box indicates mutated sequence, which was confirmed by Nanopore sequencing. *wnt7ab* mutants also had an ~8.5 kb deletion that extended into exon four. **(C, D)** Comparison of maximum average fluorescence of the TgKI(*lmx1bb*:DsRed) knock-in allele in the dorsal fins of *wnt7aa;wnt7ab* wild-type and mutant allelic combinations at standard lengths (SLs) of 6.2–7.2 mm. In (C), dorsal fins are shown in lateral view, anterior is left. No difference in maximum average fluorescence was observed. Maximum average dorsal fin fluorescence was compared using a Kruskal–Wallis test with Dunn’s multiple-comparisons test. Underlying data are provided in S1 Data. ns, not significant (*p* > 0.05).
(TIF)

S6 Fig *LARM1-LARM2* deletion results in bi-ventralized skeletal patterning of the pelvic fins and loss of pelvic splint. **(A–C)** Digital renderings of the pelvic fin skeleton from microcomputed tomography scans for *LARM1-LARM2*^+/+^ (A), *LARM1-LARM2*^*+/–*^ (B) and *LARM1-LARM2*^*–/–*^ (C) fish. Segmentation-based reconstruction of the medial-most fin ray is shown in blue both overlain on the fin (A–C) and in isolation (A’–C’). **(D, E)** Fin ray schematic (D) indicating positions of measurements used for quantifications (E). No differences between fin ray width below split of the dorsal and ventral hemitrichia (“lower” width) were observed among genotypes (Kruskal–Wallis with Dunn’s post hoc, all adjusted *p* > 0.05). Neither the width of separation between the distal margins of the dorsal (dh) and ventral hemitrichia (vh) (upper width), nor the upper/lower width ratios, differed between *LARM1-LARM2*^+/+^ and *LARM1-LARM2*^+/*–*^ genotypes. However, both the upper width and the ratio of upper/lower width were significantly different in *LARM1-LARM2*^*–*/*–*^ mutants (one-way ANOVA with Tukey’s multiple-comparisons test, **, *p* = 0.0017; and *** *p* < 0.001). **(F–I)** Digital renderings of the partial pelvic fin skeleton from microcomputed tomography scans for *LARM1–LARM2*^*+/+*^ (F), *LARM1–LARM2*^*+/–*^ (G, H), and *LARM1–LARM2*^*-–/–*^ (I) fish. * indicates the absence of the pelvic splint (plvsp). **(J)** Pelvic splint (plvsp) presence and absence were compared across genotypes using a Fisher’s exact test. Spine presence differed by genotype (*p* = 0.0105); pairwise testing showed WT differed from homozygous mutants (**, *p* = 0.0079), while WT versus heterozygotes (*p* = 0.2081) and heterozygotes versus homozygotes (*p* = 0.1049) were not significant. Underlying data are provided in S1 Data.
(TIF)

S7 Fig *lmx1bb* homozygous null mutants are embryonic lethal. **(A)** CRISPR/Cas9-mediated introduction of a premature STOP codon (red *) in the third exon of *lmx1bb*. Sanger sequencing confirmed mutation. **(B)** Dorsal and lateral views of 7dpf *lmx1bb*^*+/+*^*, lmx1bb*^*+/–*^*, lmx1bb*^*–/–*^ zebrafish. Scale bar = 500 µm.
(TIF)

S1 Table Fluorescent in situ hybridization probe sets for zebrafish. (XLSX)

S1 Data Underlying data for Figs 4D, S5D, and S6E, S6J. Each tab of the spreadsheet corresponds to a single figure panel. Values are provided for each individual fish. Abbreviations: AF, anal fin; DF, dorsal fin; PCT, pectoral fin; PLSP, pelvic splint; PLV, pelvic fin.
(XLSX)

## Abbreviations

dh: dorsal hemitrichia
dm: dorsal muscle mass
DV: dorsoventral
FISH: fluorescent in situ hybridization
GFP: green fluorescent protein
mCh: mCherry
µCT: micro-computed tomography
SLs: standard lengths
vh: ventral hemitrichia
vm: ventral muscle mass.
